# Nusinersen for type-III spinal muscular atrophy: a 12-month retrospective study in a Brazilian cohort

**DOI:** 10.1055/s-0046-1824578

**Published:** 2026-07-17

**Authors:** Vanessa Van Der Linden, Alessandra Paula de Melo Calado, Gabriela Van Der Linden, João Vitor Duque Porto Valença, Mateus Alves Garrido, Karina Lúcia Soares de Oliveira, Marcelo Soares Kerstenetzky, Thiago Oliveira

**Affiliations:** 1 Hospital Maria Lucinda, Serviço de Referência em Doenças Raras (Rarus), Recife PE, Brazil.; 2 Hospital Barão de Lucena, Recife PE, Brazil.; 3 Centro Universitário Maurício de Nassau (Uninassau), Escola de Medicina, Recife PE, Brazil.; 4 Faculdade Pernambucana de Saúde, Escola de Medicina, Recife PE, Brazil.; 5 Universidade Federal de Pernambuco, Hospital das Clínicas, Recife PE, Brazil.

**Keywords:** Spinal Muscular Atrophies of Childhood, Drug Therapy, Treatment Outcome

## Abstract

**Background:**

Spinal muscular atrophy (SMA) is a progressive, autosomal recessive motor neuron disorder caused by mutations in the *SMN1* gene. While clinical trials in type-I and -II SMA led to the approval of nusinersen for all SMA types, evidence on its long-term efficacy and safety in type-III patients is still scarce.

**Objective:**

The present study investigated treatment responses in SMA type-III patients subjected to nusinersen therapy.

**Methods:**

Pediatric and adult patients with genetically confirmed 5q SMA type III on nusinersen treatment for at least 12 months were included in the current retrospective study. We collected data on demographics, genetics, nutritional status, swallowing function, and adverse events associated with nusinersen treatment. Motor function was assessed in all patients using the Hammersmith Functional Motor Scale-Expanded (HFMSE), and respiratory function was evaluated through forced vital capacity in patients aged 6 and older.

**Results:**

Nineteen patients (12 males; 63.2%) were included in the study. Most patients (94.7%) had a homozygous *SMN1* exon 7 deletion, and 63.2% had 3 *SMN2* copies. At baseline, 68.4% of patients were ambulant, 21.1% had scoliosis, and 36.8% had lower limb deformities. One patient required non-invasive ventilation, which was discontinued after treatment with nusinersen. Hammersmith Functional Motor Scale-Expanded scores improved in 94.7% of patients after 12 months of treatment, with gains of 1 to 9 points (mean 4.0 points; *p* < 0.001). Adverse events were mild and resolved within 24 hours.

**Conclusion:**

Nusinersen was well tolerated and improved motor function in SMA type-III patients, supporting its potential benefits in real-world settings.

## INTRODUCTION

Spinal muscular atrophy (SMA) is a hereditary autosomal recessive disease characterized by the selective degeneration of motor neurons in the spinal cord and brainstem, leading to progressive muscle weakness and atrophy. 1 The disease is caused by homozygous deletion or mutation of the survival motor neuron ( *SMN* ) 1 gene on chromosome 5q13, 2 with disease severity modulated by the number of *SMN2* copies. 3 4 *SMN1* produces full-length, functional SMN protein, whereas *SMN2* has a single nucleotide substitution (840C→T) in exon 7, leading to exon-7 exclusion in most transcripts. This results in the production of a truncated, unstable form of SMN protein, limiting *SMN2* 's ability to compensate for the loss of *SMN1* function. 2 Despite this limitation, having multiple *SMN2* copies can partially mitigate disease severity by increasing the overall amount of functional SMN protein. 3 4

Type-III SMA usually manifests after 18 months of age, typically between 2 and 17 years, allowing affected individuals to achieve independent ambulation before symptom onset. 5 The disease follows a progressive but slow course, with muscle weakness and atrophy primarily affecting the proximal lower limbs and later spreading to the upper limbs and trunk. 5 It is clinically heterogeneous, but patients often experience difficulty running, climbing stairs, and standing from a seated position, with some eventually losing the ability to walk, particularly those with disease onset before 3 years of age. 5 Children with type-III SMA generally do not experience respiratory, visceral, or bulbar symptoms, which are common in SMA types I and II. 5 6 7 8 Moreover, the disease does not affect cardiac function or cognitive abilities. 5 Type-III SMA accounts for 12 to 20% of SMA cases, making it less frequent than types I and II but more common than the rarer adult-onset type-IV SMA. 9

In 2016, the approval of nusinersen, the first disease-modifying treatment for 5q-SMA, represented a major breakthrough in SMA care. Nusinersen is an antisense oligonucleotide that targets *SMN2* pre-mRNA splicing, promoting the inclusion of exon 7 and, thereby, increasing the production of full-length, functional SMN protein. 10 This mechanism helps stabilize or improve motor function by compensating for the loss of *SMN1* . Nusinersen is administered intrathecally following a structured dosing regimen: 4 loading doses—3 at 14-day intervals (days 0, 14, and 28) and a 4th on day 63—followed by maintenance doses every 4 months. 11

The availability of nusinersen and other emerging therapies has shifted SMA treatment goals from symptom management to long-term functional improvement and disease stabilization. However, because most clinical trials have focused on infants and young children with severe SMA phenotypes, data on the long-term effects of nusinersen in type-III SMA remain limited, particularly in real-world settings. This has created a knowledge gap regarding its impact on older pediatric and adult patients, whose disease progression is more variable. The present study describes a cohort of type-III SMA patients treated with nusinersen. The study aims to document patient trajectories, characterize clinical responses utilizing validated assessment scales, and investigate factors that may influence treatment outcomes.

## METHODS

### Ethical aspects

All procedures in the current study followed the ethical standards of the 1964 Declaration of Helsinki and its later amendments. The research ethics committee at Faculdade Pernambucana de Saúde, Recife, Brazil, approved this study under protocol number 7.556.247 and certificate of presentation of ethical appreciation number 87433025.9.0000.5569. Patients provided informed written consent to be part of the study, and their personal details were removed from the work.

### Study design and patient selection

A retrospective evaluation was conducted by analyzing the medical records of patients diagnosed with 5q type-III SMA and followed at the Reference Center for Rare Diseases (Serviço de Referência em Doenças Raras, Rarus, in Portuguese) at Hospital Maria Lucinda, in Recife, Pernambuco, Brazil. Rarus is the designated center for nusinersen treatment in Pernambuco, receiving patients referred by the Pernambuco State Health Department or other healthcare services. The sample size was determined by the availability of patients meeting the inclusion criteria within the study period (Febuary 2019–November 2024).

Patients of both sexes with a genetically confirmed diagnosis of 5q type-III SMA who had been receiving nusinersen for at least 1 year were included in the study. The diagnosis of 5q SMA was established in all patients, initially through multiplex ligation-dependent probe amplification (MLPA) deletion testing on the *SMN1* gene, and, when necessary, sequencing of the *SMN1* gene. Additionally, multiplex ligation-dependent probe amplification (MLPA) analysis of the *SMN2* gene was conducted to determine the number of copies of this gene. A diagnosis of 5q SMA was confirmed in patients with a homozygous deletion of exon 7 in the *SMN1* gene or a deletion of exon 7 in the *SMN1* gene associated with one pathogenic mutation in the *SMN1* gene. Patients with 5q type-III SMA who discontinued treatment or were unable to follow the prescribed regimen due to clinical complications or bureaucratic issues related to medication acquisition were excluded from the study.

Patients who met the inclusion criteria were evaluated by a pediatric neurologist, geneticist, speech therapist, and physiotherapist as part of their routine follow-up. The collected data included demographic and genetic characteristics, such as age, sex, *SMN1* mutation type, and *SMN2* copy number, as well as clinical history, including age at symptom onset, age at diagnosis, and baseline motor function. Treatment-related variables encompassed the duration of nusinersen therapy, treatment adherence, and reported side effects after nusinersen administration.

### Clinical evaluation

Standardized evaluations were conducted at baseline (before the first nusinersen dose), before the fourth dose, and every four months thereafter (up to the sixth dose of nusinersen) to assess motor function, respiratory capacity, nutritional status, and speech-language function. This evaluation schedule reflects the standard clinical routine established by the center in 2019 upon the initiation of treatment for patients with SMA.

#### *Motor function assessment*

Motor function was evaluated using the Hammersmith Functional Motor Scale-Expanded (HFMSE), a standardized scale designed to measure functional motor abilities in individuals with SMA who can sit, regardless of whether they are able to walk or not. The scale consists of 33 items, each scored from 0 to 2, with a total score ranging from 0 to 66 points. The HFMSE is validated for use in individuals aged two years and older. 12 All instructions for evaluation were followed as outlined in the English version of the HFMSE Manual. 13

#### *Respiratory function assessment*

Respiratory function was assessed in patients 6 years and older through forced vital capacity (FVC) measurement. Patients with significant respiratory impairment underwent further assessment to determine the need for non-invasive ventilation or lung expansion techniques. The evaluation focused on identifying hypoventilation, reduced lung capacity, and respiratory muscle weakness.

#### *Nutritional and swallowing assessments*

Nutritional status and swallowing function were evaluated to monitor oral feeding ability, weight maintenance, and potential dysphagia-related complications. Clinical assessments included patient-reported feeding difficulties and signs of malnutrition, while speech-language pathologists conducted specific examinations to detect orofacial hypotonia, tongue fasciculations, and other swallowing-related impairments.

### Statistical methods

Shapiro-Wilk was used to test for the distribution of variables. Continuous variables (interval or ratio data) were summarized using measures of central tendency and dispersion. The repeated measures analysis of variance (ANOVA) was employed to detect statistically significant differences in mean HFMSE scores across the study follow-up. When the repeated measures ANOVA indicated a significant overall effect, Tukey's post-hoc test was applied to perform pairwise comparisons between time points. The Statistica 7.0 software (TIBCO Software Inc.) was used for data analysis, and *p* -values < 0.05 were considered statistically significant.

## RESULTS

During the study period, 123 patients with a confirmed diagnosis of 5q SMA were monitored. Of these, 33 patients (26.8%) were classified as having type-III SMA. Among them, 24 patients (72.7%) started receiving treatment with nusinersen. The remaining patients were either not eligible for treatment or had not initiated therapy at the time of data collection. Of the 24 patients with type-III SMA undergoing nusinersen treatment, only 19 completed at least 1 year of regular follow-up and were included in the present analysis. The other 5 patients were excluded due to treatment interruptions, either from medication supply issues (n = 4) or because they had not yet completed one year of follow-up at the time of data collection (n = 1).

### Patients' demographic, clinical, and genetic characteristics

Table 1 summarizes the demographics and clinical characteristics of the 19 patients included in the current study. Twelve (63.16%) patients were male and 7 (36.84%) were female. The age at symptom onset varied between 3 months and 14 years, with a mean age of 4.5 years, while the mean age at diagnosis was 7.52 years (range: 1.5–20 years). Although type-III SMA is classically defined by symptom onset after 18 months of age, for patient 1, the onset of symptoms was considered at 3 months. This determination was based on the mother's retrospective observations, who noted slowness and delayed milestones from that age, even though the more clinically evident signs of weakness and functional difficulty emerged from 10 months, with walking initiated late and with difficulty at 1 year and 8 months. The mean age at the start of treatment was 10.1 years (range: 21 months–42 years).

**Table 1:** Patient demographics and clinical characteristics

Variable		N	Mean (range)
Age at symptom onset (years)		19	4.5 (0.2–16)
Age at diagnosis (years)		19	7.52 (1.5–22)
Age at treatment initiation (years)		19	15.6 (6–45)
		**N**	**%**
Sex	Female	7	36.84
	Male	12	63.16
*SMN1* alteration	Homozygous deletion	18	94.74
	Deletion and mutation (p.Gln154*)	1	5.26
Number of *SMN2* copies	2	2	10.53
	3	12	63.15
	4	5	26.32
Consanguinity	Yes	4	21.05
	No	15	78.95
Non-invasive ventilation before treatment	Yes	1	5.25
	No	18	94.75
Independent gait	Yes	13	68.42
	No	6	31.58
Scoliosis	Yes	4	21.05
	No	15	78.95
Deformities of the upper limbs	Yes	0	0
	No	19	100
Deformities of the lower limbs	Yes	7	36.84
	No	12	63.16
Oral motor dysfunction	Yes	2	10.53
	No	17	89.47
Fatigue with oral feeding	Yes	0	0
	No	19	100

Regarding genetic findings, 18 patients (94.7%) had a homozygous deletion of exon 7 in the *SMN1* gene, while 1 patient (5.3%) presented with a compound heterozygous mutation (an *SMN1* deletion on one allele and a mutation [p.Gln154] on the other allele). Analysis of *SMN2* copy number revealed that 2 patients (10.5%) had 2 copies, 12 patients (63.2%) had 3 copies, and 5 patients (26.3%) had 4 copies. Consanguinity was reported in 4 out of 19 patients (21.1%).

Non-invasive ventilation before treatment was required by 1 patient (5.3%). This patient exhibited a forced vital capacity of 48% of the predicted value before treatment and used bag valve mask-assisted lung expansion along with non-invasive ventilation during sleep. Following treatment with nusinersen, the patient showed progressive improvement in respiratory function, with forced vital capacity rising to over 60% of the predicted value, which allowed for non-invasive ventilation discontinuation. The remaining 18 patients (94.7%) did not require non-invasive ventilation before treatment or throughout the study follow-up.

Thirteen patients (68.4%) were able to walk independently at baseline, either for short or long distances, while 6 patients (31.6%) were non-ambulant. Scoliosis was observed in 4 patients (21.1%). No upper limb deformities were noted in the sample, while 7 patients (36.8%) presented with lower limb deformities, mostly hip and knee flexion contractures.

All patients were able to eat orally and did not report feeding difficulties before treatment or during the study follow-up. However, despite having no complaints, 2 patients (10.5%) showed abnormalities during the clinical evaluation conducted by the speech-language pathologist, presenting either tongue hypotonia or significant tongue fasciculations.

### Motor function assessment

**Figure 1:**
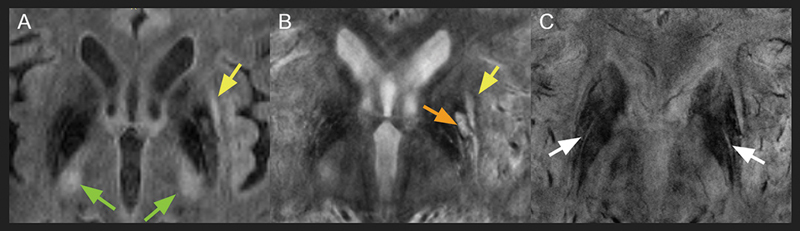
Note: * *p* < 0.01 compared with baseline (before the first dose of nusinersen). Changes in Hammersmith Functional Motor Scale—Expanded scores over time in type-III SMA patients treated with nusinersen. Statistical analysis was performed by repeated measures analysis of variance, followed by Tukey's post-hoc test. Each point represents the mean ± standard error of the mean (SEM) of 18 patients.

All patients who completed at least 1 year of regular treatment underwent motor function assessment using the HFMSE. As shown in Table 2 , none of the patients experienced a decline in their HFMSE scores during this period. Eighteen patients (94.7%) improved their HFMSE scores, with gains ranging from 1 to 9 points. Only 1 patient (5.3%) did not show a change in the score but maintained their baseline motor function. The repeated measures ANOVA revealed a statistically significant difference in the mean HFMSE scores over time (F (3, 51) = 27.33; *p* < 0.001) ( Figure 1 ). Post-hoc analysis indicated a significant increase in mean HFMSE scores from the first evaluation (before the first ^t^ dose) to all subsequent time points. After the fourth dose, motor function gains plateaued, with no significant differences observed in the assessments conducted up to the sixth dose.

**Table 2:** Changes in HFMSE scores after one year of nusinersen treatment

Patient	Deambulant	Age (years) at treatment initiation	HFMSE score				HFMSE score change
			Before 1 ^st^ dose	Before 4 ^th^ dose	Before 5 ^th^ dose	Before 6 ^th^ dose	
1	Yes	1.75	39	45	45	44	5
2	Yes	42	34	37	38	37	3
3	Yes	5	54	56	56	55	1
4	No	4	35	41	41	41	6
5	Yes	16	55	58	57	59	4
6	Yes	10	63	63	64	64	1
7	Yes	5	56	59	63	63	7
8	Yes	18	62	62	63	62	0
9	No	14	45	52	53	53	8
10	Yes	13	46	49	52	54	8
11	Yes	18	45	48	48	47	2
12	No	13	35	38	38	38	3
13	No	7	32	–	34	36	4
14	Yes	4	45	47	49	54	9
15	Yes	10	41	42	42	42	1
16	No	18	17	23	23	24	7
17	Yes	6	53	56	58	57	4
18	Yes	9	62	62	63	63	1
19	No	10	31	32	33	33	2

Abbreviation: HFMSE, Hammersmith Functional Motor Scale—Expanded.

Additional follow-up assessments were conducted for a subset of patients beyond the sixth dose. Fourteen patients underwent HFMSE evaluation before the secenth dose of nusinersen, showing a mean gain of 0.6 points (range: −4 to 3) compared to their scores before the sixth dose. Thirteen patients underwent motor function evaluations before both the eighth and ninth doses, showing mean gains of 1.53 points (range: −1 to 5) and 1 point (range: −3 to 5), respectively, compared to their scores before the sixth. Twelve patients were assessed before the tenth dose, with a mean gain of 1 point (range: −3 to 8) in the HFMSE compared to their scores before the sixth dose. Ten patients completed evaluations before the eleventh and twelfth doses, with mean gains of 1.1 points (range: −4 to 8) and 0.9 points (range: −4 to 9), respectively, relative to their sixth dose assessment. Three patients had motor assessments before the thirteenth and fourteenth doses, showing mean gains of 3.25 points (range: −1 to 10) and 4 points (range: 0–9), respectively, compared to their scores before the sixth. One patient completed evaluations before both the fifteenth and sixteenth doses, with gains of 0 and 1 point, respectively, when compared to the HFMSE score obtained prior to the sixth dose.

### Adverse events

Nusinersen treatment was generally well tolerated among the 19 patients included in the study. Nine patients (47.4%) experienced at least one post-administration adverse event; headache was the most frequently reported adverse event, occurring in 7 (36.8%) of the 19 patients. The majority of the patients affected (n = 6/7) experienced only a single episode of headache. Only one patient (n = 1/7) presented with recurrent headaches, totaling three episodes across different administrations. All observed adverse events were transient, self-limiting, and did not necessitate hospitalization.

Pain at the puncture site was also reported, with two episodes noted in one patient. Additionally, isolated cases of lower back pain (2 patients), leg pain (1 patient), and nausea (1 patient) were recorded. All adverse events were transient and resolved spontaneously within 24 hours. Notably, no serious adverse events or complications related to the administration of nusinersen were observed during the study period.

## DISCUSSION

The present study analyzed 19 patients with genetically confirmed 5q type-III SMA who completed at least 1 year of nusinersen treatment. The sample included 12 males (63.2%) and 7 females (36.8%), reflecting a slight male predominance in disease occurrence in our cohort. Although SMA is typically considered sex-neutral, some registry data suggest higher male vulnerability, 14 15 highlighting the need for further research on sex differences in SMA epidemiology.

The age at symptom onset ranged from 3 months to 14 years, with a mean of 4.5 years. This wide variability in symptom onset mirrors findings from other cohorts, with type-III SMA exhibiting a broad clinical spectrum, with symptoms appearing from early childhood to adolescence. 5 The mean age at diagnosis (7.52 years) in our cohort reflects a diagnostic delay of approximately 3 years (difference between mean age at symptom onset and mean age at diagnosis), which, while significant, is notably shorter than the delay reported by Mendonça et al. (2024), 16 who found a median diagnostic delay of approximately 14 years for type-III SMA in Brazilian patients. Several factors could contribute to this difference, including increased disease awareness in specialized centers, regional variations in healthcare access, or referral patterns that facilitate earlier diagnosis in specific clinical settings. Nevertheless, the diagnostic delay in both cohorts highlights the ongoing challenges in timely recognition of type-III SMA, particularly due to its mild and heterogeneous presentation that often complicates early identification. 17 18

Patients in our cohort began nusinersen treatment at a mean age of 10.1 years (range: 21 months–42 years), which is older than in many clinical trials but consistent with real-world settings, where treatment often starts later due to delays in therapy access. 18 19 Data from the Brazilian SMA registry revealed that only 31.9% of patients with SMA type III had access to nusinersen, primarily due to public health system restrictions that limit nusinersen distribution to type-I and -II SMA patients. 16 Despite this, improvements in motor function were still observed in our sample, supporting existing evidence that nusinersen can be beneficial even when treatment begins in adolescence or adulthood. 20

Respiratory involvement in type-III SMA is generally less pronounced than in types I and II, 5 6 but it remains a concern in certain cases. In our cohort, only 1 patient required non-invasive ventilation before treatment due to reduced forced vital capacity (48% of the predicted value). This patient had symptom onset at 2 years of age, received a diagnosis at 7 years, and initiated nusinersen treatment at 13 years. Following nusinersen treatment, the patient's forced vital capacity increased to over 60% of the predicted value, enabling non-invasive ventilation discontinuation. Studies suggest that earlier initiation of nusinersen leads to better functional outcomes, including respiratory improvements. 21 22 However, this case demonstrates that even patients starting treatment later in the disease progression can experience meaningful gains.

At baseline, 13 patients (68.4%) maintained independent ambulation, either for short or long distances, while six patients (31.6%) were non-ambulant. This is consistent with the known heterogeneity of type-III SMA . 5 23 Scoliosis was identified in 4 patients (21.1%), and lower limb deformities—primarily hip and knee flexion contractures—were observed in 7 patients (36.8%). Despite the heterogeneity in baseline motor function, the cohort demonstrated notable improvements in motor abilities following nusinersen treatment. The HFMSE results indicate that the most substantial improvements occurred in the initial months of treatment, particularly between the baseline assessment (before the 1 ^st^ dose) and the 4 ^th^ dose, where a significant increase in scores was observed. After this initial improvement, the HFMSE scores plateaued, with no significant differences detected between the 4 ^th^ , 5 ^th^ , and 6 ^th^ doses. This pattern suggests that nusinersen provided early functional gains, which were subsequently maintained, indicating stabilization of motor function in the later stages of treatment. Notably, follow-up data from a subset of patients who underwent extended assessment (up to before the 16 ^th^ dose) revealed that motor function was maintained in most patients, with additional clinically relevant gains observed in specific cases. The consistent improvements in HFMSE scores across the cohort, regardless of baseline ambulatory status or the presence of orthopedic deformities, highlight the efficacy of nusinersen in enhancing or maintaining motor abilities in type-III SMA patients. These findings align with previous studies showing that nusinersen provides a 12-month treatment benefit in type-III SMA patients, irrespective of ambulatory status. 15 24 25 26 27

In our cohort, nusinersen was generally well tolerated, with adverse events reported in 9 out of 19 patients (47.4%), mainly headache and lower back pain. These findings are consistent with prior real-world studies and clinical trials that have reported post-lumbar-puncture headache and lower back pain as two common adverse events after intrathecal nusinersen administration. 27 28 29 Our findings support the favorable safety profile of nusinersen, with no new or unexpected safety concerns emerging during the 12-month follow-up period. This reinforces the notion that nusinersen remains a safe long-term treatment option for type-III SMA patients.

The current study provides valuable information into the efficacy and safety of nusinersen in type-III SMA patients; however, it has limitations. First, while the cohort offers important real-world data, its size may not fully capture the variability in treatment responses observed in larger populations. Secondly, the lack of a control group (such as untreated patients or those receiving placebo) makes it difficult to attribute observed improvements solely to nusinersen. Although natural history data suggest that type-III SMA patients typically experience a gradual decline in motor function over time, 30 31 the absence of a control group precludes definitive conclusions regarding the magnitude of nusinersen's effect. Finally, as a single-center study, the findings may not broadly apply to other settings. Differences in multidisciplinary care practices, rehabilitation protocols, and healthcare systems can influence treatment outcomes, highlighting the need for multicenter studies to validate and expand upon these findings.

The present study suggests that nusinersen is well tolerated and improves motor function in patients with type-III SMA over 12 months. This is indicated by consistent gains in HFMSE scores and the absence of functional decline, even in patients with delayed treatment initiation. Although limited by the study's retrospective design and small sample size, these findings support the potential benefits of nusinersen in stabilizing or enhancing motor function in a real-world setting. It is important to emphasize that while numerous international real-world studies on nusinersen, including for type-III SMA, are available, 32 comparable evidence from the Brazilian context is notably limited. Therefore, the current research provides a significant local perspective on the treatment's effectiveness.
